# First description of *Eucoleus garfiai* (Gallego and Mas-Coma, 1975) in wild boar (*Sus scrofa*) in Italy

**DOI:** 10.1007/s00436-022-07505-8

**Published:** 2022-04-01

**Authors:** Laura Pacifico, Maria Francesca Sgadari, Nicola D’Alessio, Francesco Buono, Brunella Restucci, Giovanni Sgroi, Martina Ottaviano, Martina Antoniciello, Alessandro Fioretti, Claudia Tamponi, Antonio Varcasia, Vincenzo Veneziano

**Affiliations:** 1 Department of Veterinary Medicine and Animal Productions, Università Degli Studi Di Napoli “Federico II”, Naples, Italy; 2 Istituto Zooprofilattico Sperimentale del Mezzogiorno, Portici, Italy; 3 Osservatorio Faunistico Venatorio - Regione Campania, Naples, Italy; 4 Department of Veterinary Medicine, University of Bari Aldo Moro, Valenzano, Italy; 5 Department of Veterinary Medicine, University of Sassari, Sassari, Italy

**Keywords:** *Capillaria garfiai*, Italy, Wild boar, Swine

## Abstract

*Eucoleus garfiai* (syn. *Capillaria garfiai*) is a nematode infecting lingual tissue of domestic and wild swine. Prevalence data for this parasite are scant and often related to accidental findings, occurring only in Japan and a few European countries. In this study, an epidemiological survey was performed in order to identify *E. garfiai* in wild boar from the Campania region, southern Italy. A total of 153 wild boar carcasses were inspected over the course of two hunting seasons (2019–2020). Histological examinations were performed on tongue samples fixed and stained with haematoxylin and eosin. The scraping of dorsal tongue tissue was carried out to collect adult worms for parasitological examination. Out of 153 wild boars, 40 (26.1%, 95% *CI*: 19.8–33.6%) tested positive for helminths and/or eggs in tongue tissues. Parasites were identified morphologically and identification was confirmed by molecular analysis of the 18S rRNA gene, showing a 99% nucleotide match with *E. garfiai* sequences available in literature. No statistically significant differences were found according to age, sex nor hunting province. Our findings agree with previous histopathological data confirming the low pathogenic impact of this nematode. The present study represents the first report of *E. garfiai* in wild boar from Italy.

## Introduction

*Eucoleus garfiai* (syn. *Capillaria garfiai*) (Gállego and Mas-Coma 1975) is a nematode infecting lingual tissue of domestic and wild swine (*Sus scrofa*). Female parasites are mainly localized in the first quarter of the tongue and, in decreasing order, the second, third and fourth, while males are most frequently found in the third tongue quarter (Löwenstein and Kutzer 1993).

The life cycle of this parasite is, till this day, partly unknown. Experimental research has demonstrated infection in Suidae to occur through the ingestion of earthworms, such as *Lumbricus terrestris*, *Allolobophora caliginosa* and *Allolobophora* *rosea*, that function as intermediate hosts (Löwenstein and Kutzer 1993). However, it is still unclear if eggs released by adult female worms in the tongue epithelium are shed only through the faeces of the host, as reported by Ferrer and Castellà (1996) and by Masuda et al. (2019).

*Eucoleus garfiai* is recognized as a non-pathogenic parasite despite that the presence of inflammatory cells and pathological changes in the tongue tissues of infected animals has been highlighted (Ferrer and Castellà 1996; Masuda et al. 2019).

Gállego and Mas-Coma (1975) provided the first report of *E.* *garfiai* by describing the presence of a thin worm upon histological examination of tongue tissue from a wild boar from Spain. Authors presented a morphological description from worm fragments and dedicated the name of this nematode to Dr. Antonio Garfiai who performed the histological diagnosis. A detailed morphological description of the adult parasite was provided in a second study performed by Gállego et al. (1977). Two following studies, conducted in Austria by Löwenstein and Kutzer (1989, 1993), highlighted details regarding the parasite’s biological cycle. To date, *E. garfiai* has been reported, mainly as accidental findings (Ferrer and Castellà 1996), in domestic and wild swine from Japan as well as in few European countries (Masuda et al. 2019).

In Italy, as in Europe, the increasing population density of wild boar in combination with the growing public health concern towards zoonotic pathogens of this ungulate (Sgroi et al. 2019) has led to a greater interest in wild boar parasite presence and prevalence. Therefore, considering the complete lack of information on this nematode species, an epidemiological study was carried out to investigate the presence of *E. garfiai* in wild boar in Italy.

## Material and methods

### Study area and animals

Over the course of two consecutive hunting seasons (October-December 2019–2020), a total of 153 wild boar carcasses were inspected within the context of the health plan “Piano Emergenza Cinghiali in Campania” from four different hunting districts (ATCs) located in the Campania region, Southern Italy. Twenty-six samples were collected from the Avellino province (40° 54′ 55″ N-14° 47′ 22″ E) (ATC AV), 19 from Benevento (41° 08′ N-14° 47′ E) (ATC BN), 18 from Caserta (41° 10′ N-14° 13′ E) (ATC CE) and 90 from Salerno (40° 40′ 50″ N-14°45′ 35″ E) (ATC SA).

The study region has a typical Mediterranean temperate climate along the coast which transitions progressively into a continental climate inland and towards mountainous territories.

### Histological and parasitological examinations

The entire tongue of each animal was isolated and divided into four quarters. A 2-cm sample was dissected from the second quarter, over the entire thickness of the muscular organ, fixed in 10% neutral buffered formalin and embedded in paraffin wax. Four-micrometer-thick sections were stained with haematoxylin and eosin and observed by light microscopy (Leica DM2500, Leica Microsystems GmbH).

The dorsal tongue tissue from positive wild boars was scraped in order to collect adult worms for morphological examination. Parasite isolation was performed under a stereomicroscope (Leica S9i, Leica Microsystems GmbH). The collected specimens were washed in saline, preserved in 70% ethanol and examined morphologically using a light microscope (Leica DM 750, Leica Microsystems GmbH). Worms were stored for molecular analysis following morphological examination.

### Molecular analysis

DNA of collected female (*n* = 3) worms was extracted using a G-spin™ Total DNA Extraction kit (iNtRON Biotechnology, Korea), according to the manufacturer’s instructions. A fragment of 600 bp of the 18S rRNA gene was amplified using the primers 1F′and 536R (Bell and Grassle 1997; Masuda et al. 2019). Briefly, PCRs were performed in a total reaction volume of 25 μL, containing 10 × PCR buffer, 1.5 mM MgCl_2_, 0.4 mM of each deoxynucleotide triphosphate (dNTPs), 0.4 µM of each primer, 1U of Thermus aquaticus DNA Polymerase (Thermo Fisher Scientific, Massachusetts, USA) and 5 μL of DNA template. The thermal cycler conditions were 94 °C for 2 min, 40 cycles of 94 °C for 30 s, 53 °C for 30 s, 72 °C for 60 s and a final extension of 72 °C for 4 min. After electrophoresis on a 1.5% agarose gel, amplicons were purified using NucleoSpin Gel and a PCR Clean-up kit (Macherey Nagel, Germany) and sequenced by an external sequencing service (Eurofins Genomics, Germany). Samples were sequenced in both directions using the same primers as for the PCRs. The consensus sequences obtained were edited using BioEdit software (version 7.2) and compared to those available in the GenBank database through the Basic Local Alignment Search Tool (BLAST; blast.ncbi.nlm.nih.gov/Blast.cgi).

### Statistical analysis

A chi-squared test (*χ*^2^ was performed in order to compare parasite prevalence according to sex (males; females) and province of origin (Avellino; Benevento; Caserta; Salerno) of the wild boars, as well as a chi-squared test for trend for comparison of parasite prevalence according to age groups (piglets < 1 year; sub adults 1–2 years; adult > 2 years). *P* values < 0.05 were considered statistically significant.

## Results

**Fig. 1:**
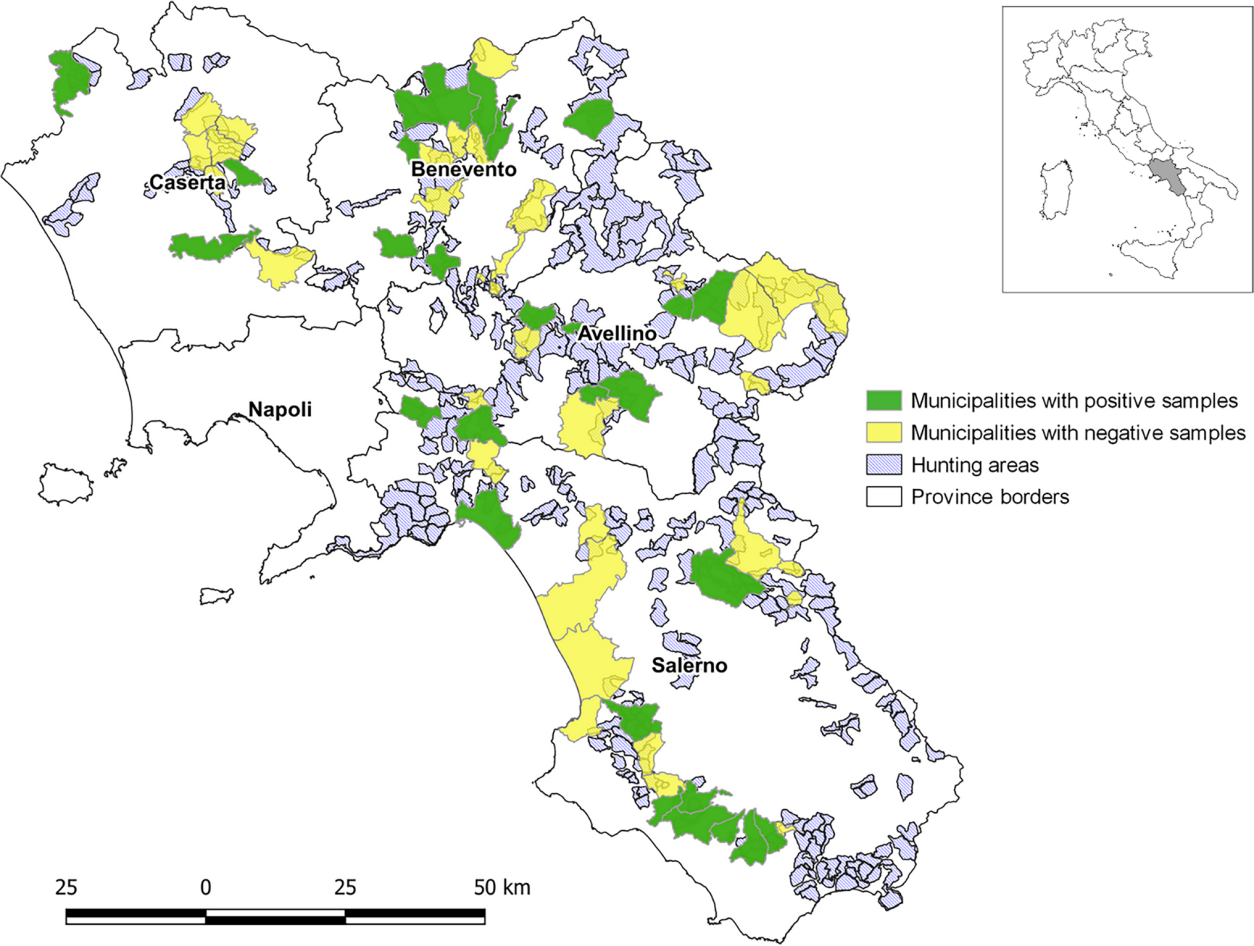
Map showing the geographical location of *Eucoleus garfiai* infected wild boar from different provinces (Avellino, Benevento, Caserta, Salerno) in relation to hunting areas in the Campania region

The presence of helminth sections and/or eggs in tongue tissue was revealed in 40 out of 153 wild boars examined histologically, for an overall prevalence of 26.1% (95% *CI*: 19.8–33.6%). No statistically significant differences were found according to sex, age nor hunting province (Table 1). The geographical distribution of positive wild boars is shown in Fig. 1.

**Table 1:** Assessment of association between *Eucoleus garfiai* infection rate and exposure variables recorded throughout the study

Variable	Category	*n*. positive/*n*. examined	Prevalence % (95% *CI*)	*P*	*OR*
Province	Caserta	4/18	22.2 (3.0–41.4)	0.59	1.00
	Salerno	21/90	23.3 (14.6–32.1)		1.06
	Avellino	8/26	30.8 (13.0–48.5)		1.55
	Benevento	7/19	36.8 (15.2–58.5)		2.04
Age (years)	Piglets (< 1)	2/10	20.0 (0–44.8)	0.90	1.00
	Sub-adult (1–2)	16/53	30.2 (17.8–42.5)		1.73
	Adult (> 2)	20/73	27.4 (17.2–37.6)		1.51
	ND	2/17	11.8 (0–27.1)		
Sex	Female	18/70	25.7 (15.5–36)	0.85	0.92
	Male	21/77	27.3 (17.3–37.2)		0.44
	ND	1/6	16.7 (0–46.5)		

*CI*, confidence interval; *P*, *P*-value; *OR*, odds ratio;* ND*, not determined

**Fig. 2:**
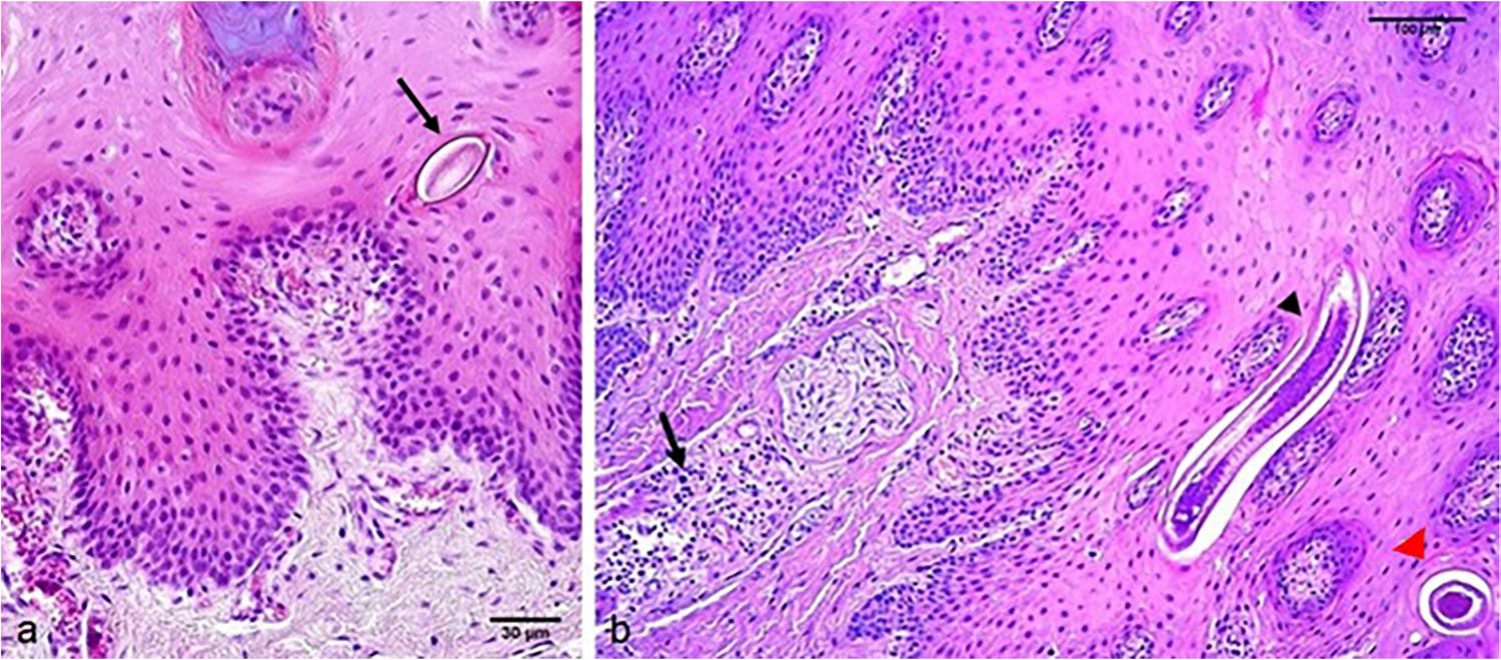
Dorsal lingual epithelium: **a** unembryonated egg (arrow) with bipolar plugs present in the hyperkeratotic epithelium. 20 × HPF; **b** worm in longitudinal section (black arrow head) and in transversal section (red arrow head) embedded in the epithelium showing hyperkeratosis of corneal layer and hyperplasia of basal layer cells. A moderate number of lymphocytes infiltrate is visible in the lamina propria of epithelium (arrow) 10 × HPF. Samples stained with haematoxylin and eosin, HE

Adult nematodes were observed in the basal layer of the lingual epithelium, while eggs were found both in the corneal layer and in prickle cell layers (Fig. 2). Macroscopic observation of infected tongue did not reveal any lesions, proving that the presence of these helminths does not cause visible alterations.

Histological evaluation revealed hyperkeratosis of the corneal layer and moderate swelling of prickled cells. Hyperplasia of basal cells was observed around adult worms. Few lymphocytes, eosinophils and plasma cells infiltrated the epithelial lamina propria (Fig. 2). Measurements of worm fragments were performed in the transversal planes on histological sections and fragments had a mean length of 69.03 μm. Eggs were measured in a longitudinal cut plane (mean 59.50 × 29.20 μm) and were barrel-shaped with two protruding polar plugs (Fig. 2).

**Fig. 3:**
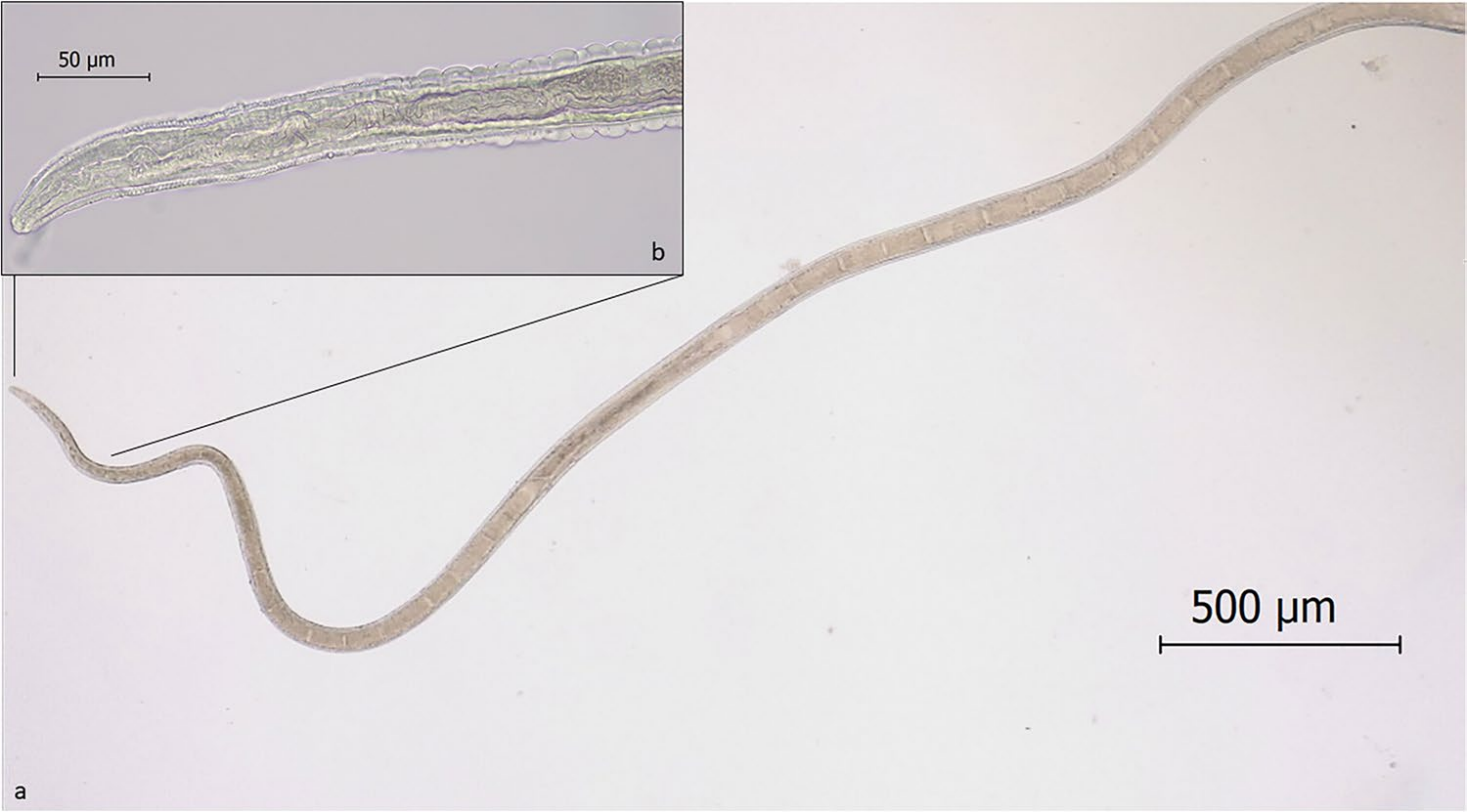
Picture of *Eucoleus garfiai* worm: (a) anterior part, with visible long stichosome and stichiocytes (4X); (b) detail of the anterior extremity, muscular oesophagus and stichiocytes (40 ×)

**Fig. 4:**
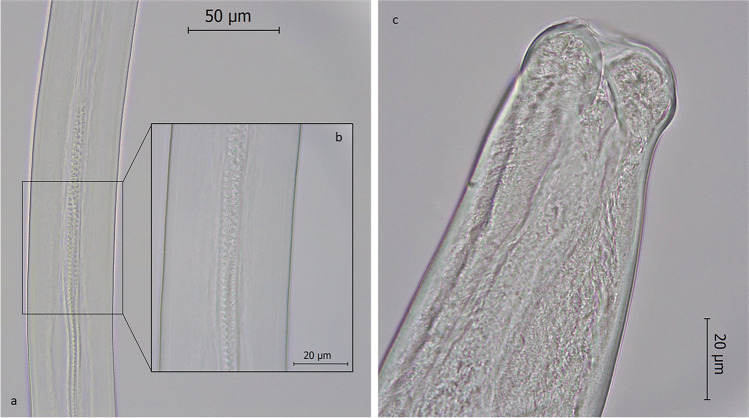
Adult male E. garfiai: (a) detail of spicule sheath (40 ×); (b) detail of spicule sheath spines (100 ×); c) posterior end (100 ×)

**Fig. 5:**
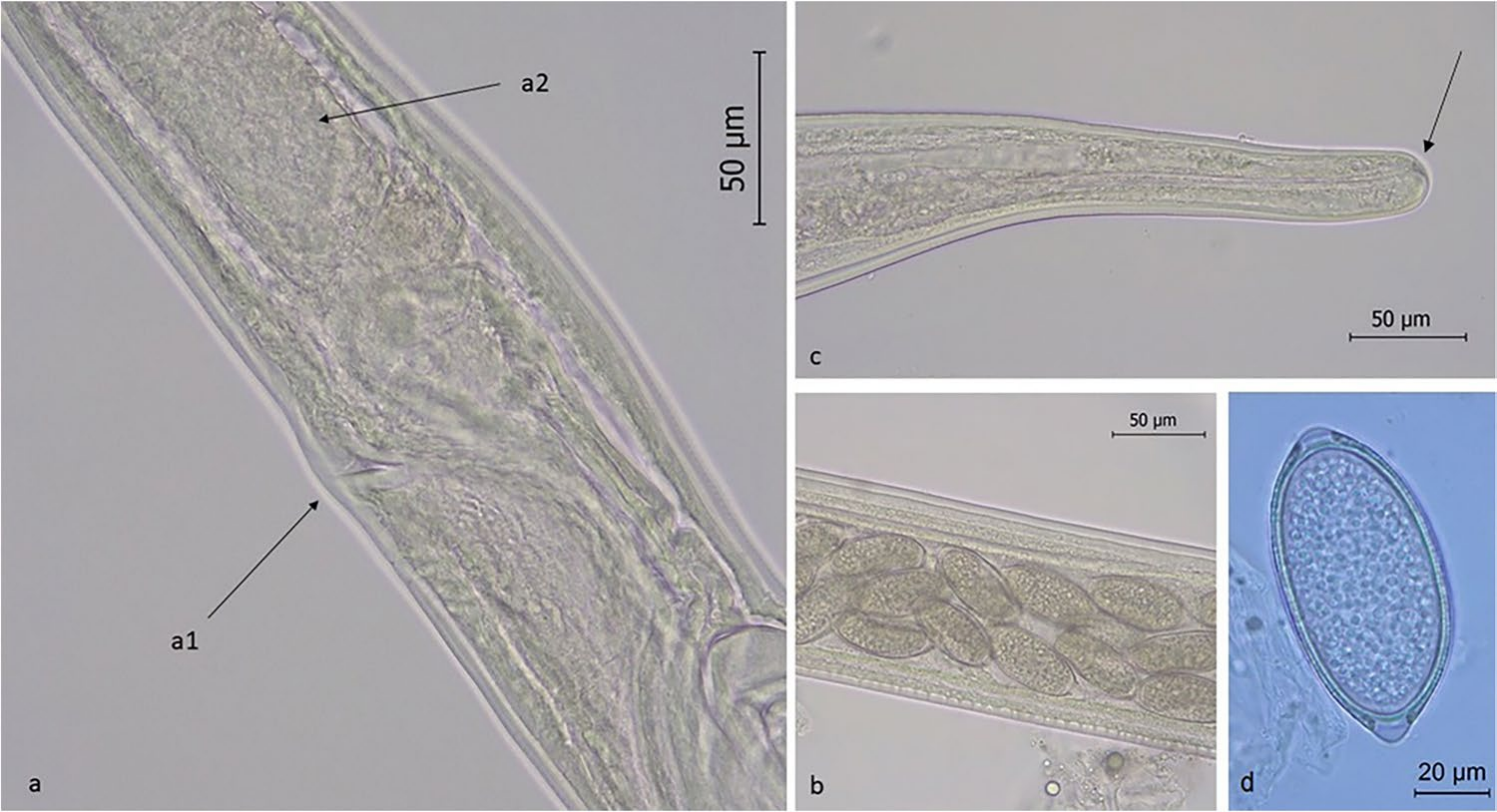
Adult female *E. garfiai*: **a** detail of vulva region (a1) and oesophagus end (a2: last stichiocyte) (40 ×); **b** detail of eggs in uterus (40 ×); **c** posterior end with visible anus (arrow) (40 ×)*;* **d** egg isolated from lingual scraping (100 ×)

Morphological examination of collected specimens revealed thin elongated worms with a long oesophagus and the presence of a stichosome. The mean number of stichocytes was 31 (range: 25–35) in females and 30 in males (Fig. 3). Male worms were 11.00 mm in length (range: 9.33–12.70 mm), 0.08 mm in width (range: 0.07–0.09 mm) and showed two caudal lateral lobes connected by a membrane. Their spicule sheath measured 1.16 mm in length and was covered in spines (Fig. 4). Female were 15.40 mm in length (range: 14.90–15.80 mm) and 0.10 mm in width (range: 0.09–0.12 mm), showing a short distance between the end of the oesophagus and vulva (mean 0.05 mm; range: 0.04–0.06 mm). Their caudal extremity was rounded with a sub-terminal anus and the uterus contained a large amount of unembryonated, barrel-shaped eggs with two polar plugs (Fig. 5). Eggs measured 54.33–66.81 × 24.76–32.12 μm (mean 61.04 × 27.22 μm).

The morphological features of the parasite herein isolated, as well as its localization and histopathological characterization, are consistent with the description of *E. garfiai* proposed by Gállego and Mas-Coma (1975) and subsequent descriptions (Ferrer and Castellà 1996; Masuda et al. 2019).

Molecular analyses of partial 18S rRNA gene showed a nucleotide identity of 99% with *E. garfiai* sequences deposited in GenBank (accession number MW947272), confirming morphological identification of the isolates.

## Discussion

The present research constitutes the first report of *E. garfiai* in Italy and the first molecular data of this parasite from wild boar in Europe.

Our findings agree with histopathological features described in previous studies, confirming the low pathogenic impact of this nematode (Ferrer and Castellà 1996; Masuda et al. 2019).

Moreover, the presence of eggs in the corneal layer of the tongue of infected animals would confirm the hypothesis that these are excreted through faecal shedding following ingestion by the host, as previously suggested (Masuda et al. 2019).

The prevalence of *E. garfiai* in wild boar herein detected is lower than that outlined in analogous epidemiological studies performed in other European countries (82%, Spain: Gállego et al. 1977; 69–90.9%, Austria: Löwenstein and Kutzer 1989). Our findings are similar to those reported in a recent study based on histological examination conducted outside the European continent, showing a prevalence of 36.4% in the Japanese wild boar (*Sus scrofa leucomystax*) (Masuda et al. 2019). However, as reported by Masuda et al. (2019), the actual infection rate in wild boar in the examined area could be higher than that reported here since these results are based on histological examination of a small sample of the tongue tissue, suggesting the importance of more sensitive molecular tools for the diagnosis of this infestation. The lack of reports of *E. garfiai* is therefore probably due to the difficulty of finding this thin nematode, which is threaded into the lingual epithelium.

The presence of this nematode in wild boar could be more common that is currently known, especially when feeding habits of these animals are taken into account. In fact, the rooting activity, which is a common behavioural feature of wild boars, allows for an easy ingestion of small invertebrates, such as earthworms, that function as intermediate hosts for *E. garfiai* (Massei and Toso 1993).

The presence of *E. garfiai* in multiple subspecies of wild boar (i.e., *Sus scrofa leucomystax* and the European wild boar, *Sus scrofa scrofa*) living in two opposite parts of the world suggests a wide adaptability of this parasite to different climatic conditions and feeding habitats. This aspect would indicate a broad distribution for the species, even in areas where the European wild boar was previously imported for breeding/hunting contexts (Rivero et al. 2013). The spreading of *E. garfiai* could also be favoured in areas where an overpopulation of wild boar occurs. In fact, wildlife overpopulation has been assessed as one of the main determinants in increased transmission pathways of parasitic infections, some of zoonotic concern (Gortázar et al. 2006). Furthermore, Navarro-Gonzalez et al. (2013) report that the habit of wild boars to defecate in the proximity of feeding places could be a risk factor for parasite infection in confined and uncontrolled populations. It is generally accepted that chances of encountering an infected intermediate host (like earthworms) are greater in feeding areas, as was demonstrated in the case of swine lungworms (e.g. *Metastrongylus* spp.) (Humbert and Henry 1989). Other factors related to human wildlife management, such as animal translocations (Fernandez-de-Mera et al. 2004), the aggregation of wild boar in fenced hunting properties and the supplemental feeding of animals (Gortázar et al. 2006; Navarro-Gonzalez et al. 2013), can influence the spread of parasites as well.

Morphologically, eggs of *E. garfiai* closely resemble those of swine infecting *Capillaria* species. Similar eggs are commonly reported as *Capillaria* sp. in various epidemiological studies (de-la-Muela et al. 2001; Moretta et al. 2011; Petersen et al. 2020; Spieler and Schnyder 2021). As suggested regarding *Capillaria aerophila*, *C. boehmi* and *Trichuris vulpis* in dogs, it would be appropriate to carry out molecular and/or egg-shell morphological differentiation in order to avoid misdiagnosis (Di Cesare et al. 2012).

The possibility of infection by *E. garfiai* through the administration of infected earthworms has also been reported in domestic pigs (Löwenstein and Kutzer 1993; Masuda et al. 2019). Moreover, although no macroscopic lesions of the tongue were observed in this study; it could be interesting to evaluate the impact of infection on food intake and weight gain in domestic pigs reared outdoors in areas potentially shared with wild boar.

In this regard, considering the wide distribution of earthworms, more attention should be paid to this Capillariidae in swine reared outdoors and in case of animal translocation in order to reduce the risk of parasite spreading (Fernandez-de-Mera et al. 2003; Rivero et al. 2013). Indeed, due to the current trend of converting animal production from indoor to outdoor housing, the possibility of parasite transmission from wild boars to domestic pigs could increase in pig farms with scarce biosecurity programmes (Petersen et al. 2020).

The absence of significant differences in infection prevalence in relation to age and sex is likely due to the same feeding behaviour of this ungulate regardless of age and sex (Ballari and Barrios-García 2014; Masuda et al. 2019). Considering that the genus *Eucoleus* parasitize mammals (Snyder 1989) and birds (Santoro et al. 2010), and that the members of this genus are reported in the oral cavity, oesophagus, stomach and respiratory tract of hosts (Gibbons 2000), it could be interesting to evaluate the presence of *E. garfiai* in other regions as well, as suggested by Masuda et al. (2019).

Large-scale surveys should be conducted in other European countries to better understand the epidemiology and prevalence of this poorly known parasite in pigs and wild boar populations.
